# Functional Study on Cytochrome P450 in Response to L(−)-Carvone Stress in *Bursaphelenchus xylophilus*

**DOI:** 10.3390/genes13111956

**Published:** 2022-10-27

**Authors:** Jie Chen, Xin Hao, Ruina Tan, Yang Li, Bowen Wang, Jialiang Pan, Wei Ma, Ling Ma

**Affiliations:** 1 School of Forestry, Northeast Forestry University, Harbin 150040, China; 2 Plant Science, Wageningen University & Research, 6708 PB Wageningen, The Netherlands; 3 Institute of Zoology, Chinese Academy of Sciences, Beijing 100101, China; 4 School of Art and Archaeology, Zhejiang University, Zhejiang University, Hangzhou 310028, China; 5 Center for Biological Disaster Prevention and Control, National Forestry and Grassland Administration, Shenyang 110034, China; 6 College of Pharmaceutical Sciences, Heilongjiang University of Chinese Medicine, Harbin 150040, China

**Keywords:** cytochrome P450, L(−)-carvone, *Bursaphelenchus xylophilus*, RNAi

## Abstract

*Bursaphelenchus xylophilus* (PWN) causes pine wilt disease (PWD), which is one of the most devastating pine diseases worldwide. Cytochrome P450 (CYP) catalyzes the biosynthetic metabolism of terpenoids and plays an important role in the modification of secondary metabolites in all living organisms. We investigated the molecular characteristics and biological functions of *Bx-cyp29A3* in *B. xylophilus*. The bioinformatics analysis results indicated that *Bx-cyp29A3* has a transmembrane domain and could dock with L(−)-carvone. The gene expression pattern indicated that *Bx-cyp29A3* was expressed in 0.2, 0.4, 0.6, 0.8, and 1.0 mg/mL L(−)-carvone solutions. The *Bx*-*cyp29A3* expression increased in a dose-dependent manner and peaked at 24 h of exposure when the L(−)-carvone solution concentration was 0.8 mg/mL. However, the gene expression peaked at 0.6 mg/mL after 36 h. Furthermore, RNA interference (RNAi) indicated that *Bx-cyp29A3* played an essential role in the response to L(−)-carvone. The mortality rates of the *Bx-cyp29A3* knockdown groups were higher than those of the control groups in the 0.4, 0.6, 0.8, and 1.0 mg/mL carvone solutions after 24 h of exposure or 36 h of exposure. In summary, bioinformatics provided the structural characteristics and conserved sequence properties of *Bx-cyp29A3* and its encoded protein, which provided a target gene for the study of the P450 family of *B. xylophilus*. Gene silencing experiments clarified the function of *Bx-cyp29A3* in the immune defense of *B. xylophilus.* This study provides a basis for the screening of new molecular targets for the prevention and management of *B. xylophilus*.

## 1. Introduction

Pine wood nematode (*B. xylophilus*, PWN), the most threatening forestry pest in eastern Asia and parts of Europe, causes pine wilt disease, which is a contagious and destructive disease [1,2]. In China, pine wilt disease (PWD) has occurred in 721 counties of 17 provinces and has rapidly spread westward and northward [3]. In recent years, although the government has made tremendous efforts to control PWD in China, it has still caused significant damage to Chinese forest ecosystems [4]. As chemicals are fast and efficient, the control of PWNs is currently carried out mainly by chemical applications to the trunk and soil [5,6]. With the extensive use of chemical nematocides, the risk of PWNs developing a resistance continues to increase. As a result, the mechanisms of drug metabolism in PWNs are gaining attention.

L(−)-carvone, a monoterpene insecticide, is environmentally friendly and effective in controlling pests [7]. Thus, carvone has been widely used for many years as an insecticide, fungicide, antioxidant, and plant growth regulator [8,9,10,11]. Carvone is widely used as a plant essential oil in the treatment of parasitic nematodes. Carvone completely inhibited egg-hatching in vitro and significantly reduced the number of fecal eggs, reduced the male body length, and reduced the female reproductive capacity in vivo in *Hemonchus contortus* [12]. In addition, carvone has an excellent nematocidal activity against *Trichostrongylus* spp., *Meloidogyne incognita*, and *M.* *javanica* [13,14,15,16].

Cytochrome P450 (CYP or P450) is a class of heme-thiolate proteins encoded by the cytochrome P450 gene superfamily [17]. These proteins have similar structures and functions. They are named for the complex formed by binding to carbon monoxide (CO) with the largest characteristic absorption peak at a wavelength of 450 nm [18]. They are an important component of the multifunctional oxidase family. Cytochrome P450 is widespread in all aerobic organisms, catalyzing a series of oxidative metabolism-related reactions in organisms and participating in the synthesis and metabolism of many important biological chemicals such as steroids [19,20], retinoids [21], prostaglandins [22,23], and fatty acids [24]. It also plays an important role in the detoxification of harmful exogenous substances such as drugs and pesticides [23,25]. Currently, the role of cytochrome P450 has been well-explained in pathogen drug resistance studies [26,27]. CYP306A is involved in ecdysteroidogenesis and insecticide metabolism in *Bombyx mandarina* and *B. mori* [28]. Cyp12a5 overexpression increases the nitenpyram sensitivity of *Drosophila melanogaster* [29]. CYP6AB12 drives *Spodoptera litura* to respond to lambda-cyhalothrin stress through the activation of ROS and the cap ‘n’ collar isoform C gene [30]. CYP321A8, CYP321A9, and CYP321B1 regulate the susceptibility of *Spodoptera frugiperda* to chlorantraniliprole [31]. CYP301B1 and CYP6AX1v2 may contribute to the resistance of *Nilaparvata lugens* to β-asarone [32]. CYP genes take part in the exposure of the nematode *Ascaridia galli* to benzimidazoles [33]. *Caenorhabditis elegans* CYP35 can upregulate in response to heavy metals, pesticides, anti-parasitic and anti-chemotherapeutic agents, polycyclic aromatic hydrocarbons (PAHs), nanoparticles, drugs, and organic chemical compounds [34]. In addition, cytochrome P450 genes are also important in the metabolism of endogenous chemicals; they promote pheromone production and improve adaptability [35] to environmental change [36].

For a long time, the control of parasitic nematodes has been mainly based on chemical control. With the need for ecologically sustainable developments, the development and application of environmentally friendly nematocides is a matter of urgency. Cytochrome P450, an important component of oxidative metabolism, has been relatively poorly studied in nematodes [37,38]. With the development of bioinformatics research, greater progress has been made in the functional study of cytochrome P450 in the nematode. The CyP450 gene responds to β-pinene [39] and levamisole hydrochloride [40] stress in pine wood nematodes. *CyP33C9* affects the reproduction and pathogenicity of pine wood nematodes [41]. CyP450 plays an active role in the response to exogenous stress. This is an important control target for endogenous and xenobiotic metabolism. This study further clarifies the mechanisms of resistance, promoting the development of plant-parasitic nematode control technologies.

## 2. Materials and Methods

### 2.1. Pharmaceutical Preparation and Nematode Culture

Reagent grade L(−)-carvone (99%) (C_10_H_14_O, CAS: 6485-40-1) was made available by Aladdin (Shanghai, China). The solutions were prepared in 5% dimethyl sulfoxide (DMSO) just before being used [42]. Other reagents such as an M9 buffer were purchased from Nachuan Biotech (Harbin, China).

*B. xylophilus* and *Botrytis cinerea* were provided by the Quarantine Pests Laboratory of the Northeast Forestry University. *B. xylophilus* was grown on cultivated *B. cinerea* using potato dextrose agar (PDA) plates at 25 °C for 7 days protected from light [43]. *B. xylophilus* was fed by *B. cinerea* [44,45]. The plate covered with *B. cinerea* was inoculated with pine wood nematodes and incubated for 7 days at 25 °C in the dark, then collected by a Baermann funnel [46], washed 5 times with the M9 buffer with centrifugation at 4000× *g* for 3 min, and then prepared for use [47]. All experiments were performed with 100 highly active *B. xylophilus* specimens in each sample.

### 2.2. RNA Isolation and Gene Acquisition

About 5000 *B. xylophilus* were collected in each sample by double-distilled water and were ground into a powder by a grinder after adding liquid nitrogen. The total RNA of the nematodes was extracted with RNAiso Plus (Takara, Dalian, China) [48]. With Oligo(dT)_18_ as the primer, TransGen #AE311 EasyScript One-Step gDNA Removal and cDNA Synthesis Super Mix (TransGen Biotech, Beijing, China) was used to reverse the first-strand cDNA. The double-stranded cDNA was synthesized by a random primer.

The *cyp29A2* sequence of *C. elegans* was used as the template sequence for BLAST against the genomic data of *B. xylophilus* in Wormbase (https://parasite.wormbase.org/Bursaphelenchus_xylophilus_prjeb40022/Info/Index, accessed on 4 March 2021) [49]. The homologous sequence of *cyp29A2* was identified and named *Bx-cyp29A3*. Polymerase chain reaction (PCR) primers covering the CDS of *Bx-cyp29A3* were designed (*Bx-cyp29A3*-F: 5′-ATGGCGTCGATTGTTGCGA-3′; *Bx-cyp29A3*-R: 5′-TCATCGTTCTTCCAAAATGACGGG-3′). The PCR amplification conditions included a pre-denature at 94 °C for 2 min, 35 cycles of denaturation at 94 °C for 30 s, annealing at 58 °C for 2 min, and an extension at 72 °C for 2 min with a final extension at 72 °C for 10 min. The PCR products were separated on 1.2% agarose gel containing ethidium bromide and then sent to Sangon Biotech for sequencing (Shanghai, China). The *Bx-cyp29A3* protein homologous sequences in the other nematodes were screened by the National Center for Biotechnology Information (NCBI). The sequence alignment analysis was performed by ClustalX and the conserved structural domain of the *Bx-cyp29A3* protein was predicted by the CD-Search tool (https://www.ncbi.nlm.nih.gov/Structure/cdd/wrpsb.cgi, accessed on 4 March 2021) [50]. The protein sequences were compared with the other sequences of nematodes deposited in GenBank using BLAST. A phylogenetic tree was constructed by the neighbor-joining (NJ) method and the maximum likelihood (ML) method in Mega11 software. A phylogenetic tree with 1000 times self-replicated sample was tested.

### 2.3. Structural Analysis of Bx-cyp29A3

An analysis of the physicochemical properties provided fundamental data for the study of the functional mechanisms of the target proteins [51]. The protein analysis tool ProtParam (https://web.expasy.org/protparam/, accessed on 4 March 2021) of the expert protein analysis system ExPASy was applied to analyze the physicochemical properties of *Bx-cyp29A3*, including the amino acid number, molecular weight, theoretical isoelectric point, lipid index, total number of atoms, and instability coefficient [52].

Protein hydrophobicity is important for protein stability and function. A transmembrane structural domain analysis of the proteins determined the function and role of the target protein. ProtScale (https://web.expasy.org/protscale/, accessed on 4 March 2021) [53] was used to analyze the protein hydrophobicity. TMHMM (http://www.cbs.dtu.dk/services/TMHMM, accessed on 4 March 2021) [54] was used to predict the transmembrane helical segments (TMHs) of the protein.

Protein phosphorylation is the most fundamental, pervasive, and important mechanism that regulates and controls protein activity and functions [55]. It is inextricably linked to many biological issues such as signaling, the cell cycle, growth, and development. Moreover, protein phosphorylation is biologically important to elucidate the protein function. NetPhos 3.1 Serve (http://www.cbs.dtu.dk/services/NetPhos/, accessed on 4 March 2021) [56] was applied to predict the protein phosphorylation sites and analyze the number of phosphorylation sites for serine, threonine, and tyrosine.

The spatial structure of protein is very important and beneficial to understand the executive functions determining the structure of the protein. The secondary structure of Bx-cyp29A3 was predicted by SOPMA (http://npsa-pbil.ibcp.fr/cgi-bin/npsa_automat.pl?page=npsa_sopma.html, accessed on 4 March 2021) [57] and the tertiary structure of Bx-cyp29A3 was homologously modeled using SWISS-MODEL (https://swissmodel.expasy.org, accessed on 4 March 2021) [58]. A Ramachandran diagram was constructed using Swiss-PdbViewer (https://spdbv.unil.ch/, accessed on 4 March 2021) [59].

### 2.4. Functional Characterization of Bx-cyp29A3

The gene expression levels of *Bx-cyp29A3* were measured by a fluorescence-quantitative PCR after 24 and 36 h of stress from 0.2, 0.4, 0.6, 0.8, and 1.0 mg/mL L(−)-carvone solutions, respectively. RNAi primers were designed by BioXM2.6 software based on *Bx-cyp29A3 (Bx-cyp29A3*-iF: GCTAATACGACTCACTATAGGGATCGCCGTCCAGAAGTTCAACCATC; *Bx-cyp29A3*-iR: AGTAATACGACTCACTATAGGGATCATGACAAGCCAATGCCCAAAGG) and then a transcriptional synthesis of dsRNA by a MAXIscript T7 RNA Synthesis Kit (Promega, Beijing, China). The concentration of dsRNA was measured by a UV spectrophotometer (Agilent MX3000P, Santa Clara, CA, USA) and the quality of dsRNA was measured by agarose gel electrophoresis after purification [60].

The dsRNA was diluted to 3.0 mg/mL with the M9 buffer and the PWNs were soaked for 24 h at 25 °C in darkness. The PWNs soaked in the *Bx-cyp29A3*–dsRNA solution were the treated group, the PWNs soaked in the *gfp*–dsRNA solution were the control group, and DEPC–H_2_O was the negative control group. All groups were observed under a microscope; individual dead PWNs were removed with picking needles. To monitor the uptake of dsRNA, the PWNs were soaked in a 1.5 mg/mL FITC solution for 24 h. A fluorescent microscope (ZEISS, Oberkochen, Germany) was used to take photographs of the FITC-treated nematodes.

The total RNA of the RNAi-treated and control PWNs was extracted by Trizol and examined by qRT-PCR using specific primers (*Bx-cyp29A3*-qF: TCTGCGAAGAGGTGGACACATT; *Bx-cyp29A3*-qR: CGGCGTAGAGTTGCTCCTGAA) with 28S (28S-F: GTGCGTATTCAGCCTTCTGG; 28S-R: AACCGAACACGCGACAATAG) as the internal reference base [61]. A quantitative data analysis was performed by 2^−ΔΔCt^ to test the efficiency of *Bx-cyp29A3* and that it was silenced. Healthy and active third instar PWNs from the RNAi-treated and control groups were subsequently picked with a picking needle and added to a mass concentration of 0.2, 0.4, 0.6, 0.8, and 1.0 mg/mL of L(−)-carvone solutions and treated for 24 and 36 h in the dark. The mortality rates of the RNAi-treated and control groups were counted. Each treatment was repeated three times.

### 2.5. Statistical Analysis

The statistical significance of the data was determined using a one-way analysis of variance (ANOVA) using GraphPad Prism 9.0 with a Student’s *t*-test with *p* < 0.05 as the significance threshold.

## 3. Results

### 3.1. Gene Amplification and Identification of Bx-cyp29A3

**Figure 1:**
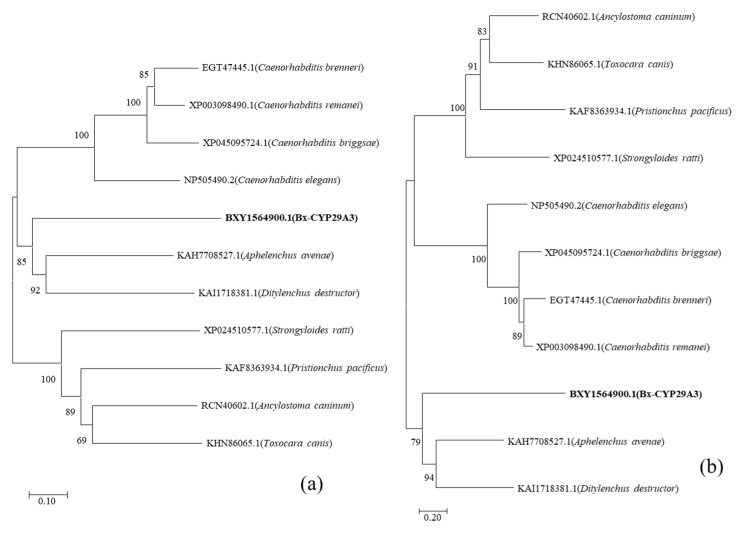
Phylogenetic analysis of *Bx-cyp29A3* with CYPs from other nematodes. (**a**) Phylogenetic tree constructed by the maximum likelihood (ML) method; (**b**) phylogenetic tree constructed by the neighbor-joining (NJ) method.

The Trizol method was used to extract the total RNA from the PWNs and the results were detected by 1.2% agarose gel electrophoresis (Figure 1a). The OD value (OD_260_/OD_280_ = 2.1, OD_260_/OD_230_ > 1.9) was determined with a GeneQuant 1300 spectrophotometer. The 28S region was twice as bright as the 18S region whereas the 5S region was darker. The extracted RNA was reverse transcribed into cDNA (Figure S1b). The cDNA obtained by reverse transcription was used as a template for the PCR amplification. The *Bx-cyp29A3* product was 1482 bp in length (Figures S1c and S2).

The homologous protein sequences of *Bx-cyp29A3* were screened at the NCBI (Table 1, Figure S3). ClustalX was applied for the multiple sequence comparisons and Mega 11 was used to construct an evolutionary tree. *Bx-cyp29A3* was clustered with *Aphelenchus avenae* and *Ditylenchus destructor* as 1 branch (1000 replicate sampling tests). Based on the results of the best protein models, the LG + G + I model (BIC = 18,962.865, AICc = 18,824.059) was selected for the evolutionary tree construction (Figure 1).

### 3.2. Structural and Functional Analysis of Bx-CYP29A3

**Figure 2:**
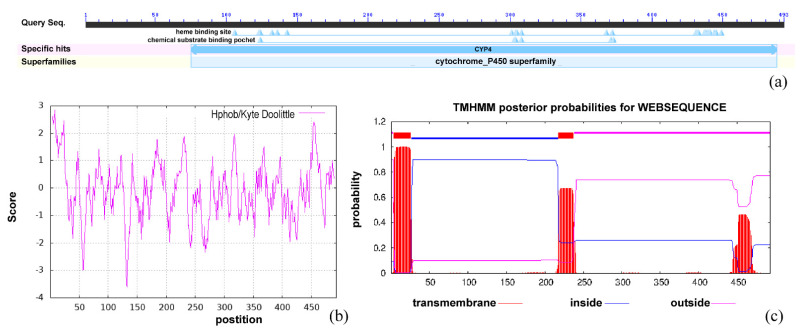
Physicochemical properties of *Bx-cyp29A3*. (**a**) Conserved domains search result of *Bx-cyp29A3*; (**b**) ProtScale analysis result of *Bx-cyp29A3*; (**c**) TMHMM server analysis result of *Bx-cyp29A3*.

*Bx-cyp29A3* was 1482 bp long and encoded *Bx-cyp29A3*, which consisted of 493 amino acids. The molecular formula of the protein was C_2602_H_4006_N_686_O_712_S_16_, the molecular weight was 56.80 kDa, the theoretical pI was 6.95, the aliphatic index was 96.73, and the grand average of hydropathicity (GRAVY) was −0.096. The instability index (II) was computed to be 38.21, which classified the protein as stable. In the *Bx-cyp29A3* protein structure, there was a highly conserved structural domain between amino acids 75 and 488; it belonged to CYP4 (cd20628, E-value = 2.83 × 10^−161^), indicating that the protein was a member of the cytochrome P450 family of proteins (Figure 2a). The hydrophobicity score of *Bx-cyp29A3* at position 133 histidine (H) was −3.622, which was more hydrophilic; the hydrophobicity score at position 8 alanine (A) was 2.844, which was more hydrophobic. The hydrophobicity analysis showed that *Bx-cyp29A3* contained multiple hydrophilic and hydrophobic regions, but the distribution was not significantly aggregated (Figure 2b). The TMHMM server and TMpred predicted that *Bx-cyp29A3* had two transmembrane regions (Figure 2c).

**Figure 3:**
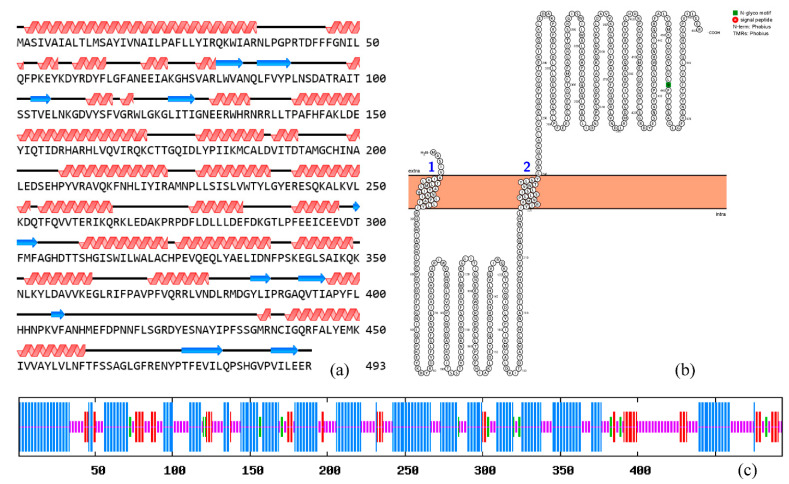
Structural analysis of *Bx-cyp29A3*. (**a**) Protein secondary structure prediction based on position-specific scoring matrices of *Bx-cyp29A3*; (**b**) Topological analysis of *Bx-cyp29A3*. Numbers represent sites of transmembrane structures; (**c**). SOPMA—Protein secondary structure prediction peak, blue: Alpha helix; Green: Beta turn; Yellow: Random coil; Red: Extended strand.

The *Bx-cyp29A3* phosphorylation site prediction indicated that this protein had 14 serine, 8 threonine, and 11 tyrosine phosphorylation sites. The secondary structure predicted that *Bx-cyp29A3* had 245 amino acids forming 29 α-helices (49.70% of the total secondary structure), 58 amino acids forming 11 β-folds (11.76% of the total secondary structure), 20 amino acids forming β-turns (4.06% of the total secondary structure), and 170 amino acids forming irregular coils (34.48% of the total secondary structure) (Figure 3a,c). A topological analysis showed that *Bx-cyp29A3* was a transmembrane protein with two transmembrane structural domains and one N-glycosylation motif (Figure 3b); thus, *Bx-cyp29A3* modified the xenobiotic metabolites.

**Figure 4:**
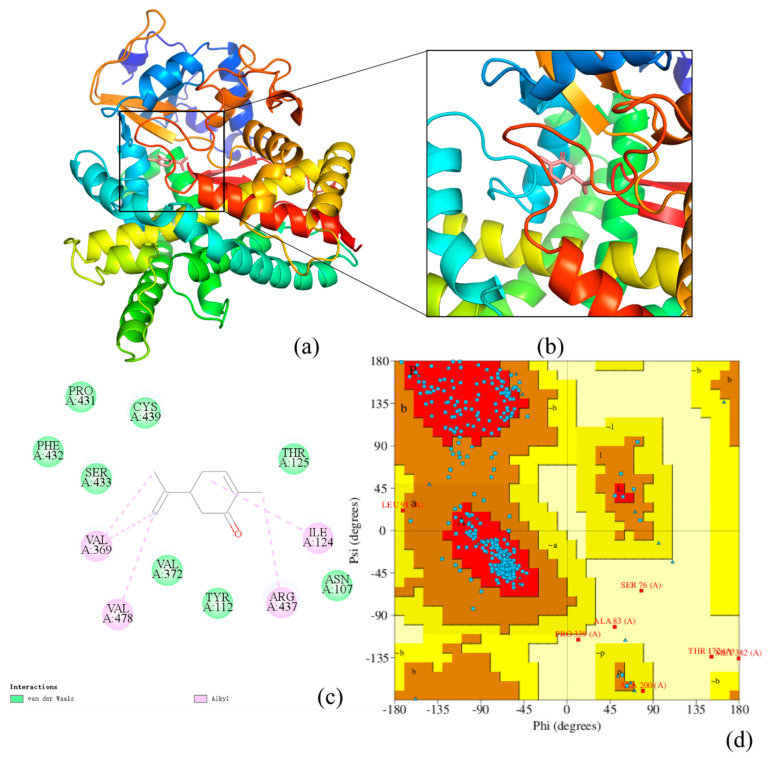
Tertiary structure prediction of *Bx-cyp29A3*. (**a**) The tertiary structural homology model of *Bx-cyp29A3*. (**b**,**c**) Molecular docking of *Bx-cyp29A3* with L(−)-carvone. The green ball represents the van der Waals forces. The pink balls represent the existence of an alkyl interaction. The pink dotted line is the σ bond formed by the alkyl force; (**d**) Ramachandran plot of *Bx-cyp29A3*. a: Core alpha; b: Core beta; L: Core left-handed alpha.

The tertiary structural homology model of *Bx-cyp29A3* by SWISS-MODEL is shown in Figure 4. The most similar model result was CYP4B1 (SMTL ID: 6c93.1. A), which showed a 30.40% similarity to *Bx-cyp29A3* [62] (Figure 4a). The three-dimensional structure of the protein showed that *Bx-cyp29A3* contained multiple amino acids involved in the α-helix and β-fold and had multiple nucleotide-binding and transmembrane structural domains. We then performed molecular docking simulations of *Bx-cyp29A3* with L(−)-carvone via Autodesk to predict the docking position (Figure 4b). The results showed that the amino acids 07ASN, 112TYR, 125THR, 372VAL, 431PRO, 432PHE, 433SER, and CYS439 were predicted to bind L(−)-carvone by a van der Waals force. The 124ILE, 369 VAL, 478VAL, and 437ARG amino acids also interacted with L-carvone through the alkyl interaction (Figure 4c). A Ramachandran plot was used to assess the three-dimensional structure of *Bx-cyp29A3* (Figure 4d). The dihedral angles of the *Bx-cyp29A3* residues were found to be located in the yellow core region and the spatial structure was found to be over 90% stable, indicating a high degree of confidence in the tertiary structure.

### 3.3. Effect of Bx-cyp29A3 Silencing on the PWN Response to L (−)-Carvone Stress

**Figure 5:**
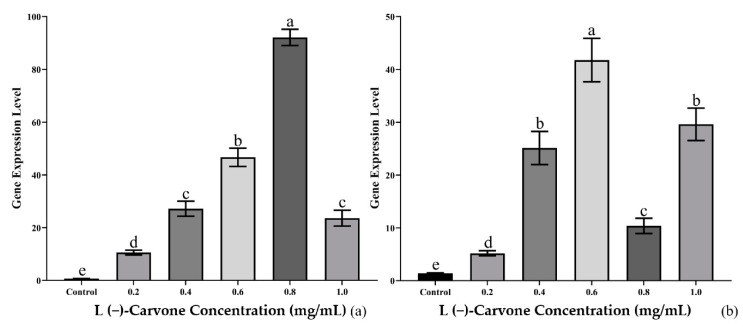
Gene expression of *Bx-cyp29A3* treated with L(−)-carvone solutions. (**a**) 24 h treatment with L(−)-carvone solutions of 0.2, 0.4, 0.6, 0.8, and 1.0 mg/mL; (**b**) 36 h treatment with L(−)-carvone solutions of 0.2, 0.4, 0.6, 0.8, and 1.0 mg/mL. Notes: Data are mean values ± SD of different repetitions; *n* = 3. Different letters indicate statistical differences with *p* ≤ 0.05 from Student’s *t*-test.

The gene expression levels of *Bx-cyp29A3* by real-time qPCR were upregulated after 24 h or 36 h of treatment with L(−)-carvone solution concentrations of 0.2, 0.4, 0.6, 0.8, and 1.0 mg/mL. The gene expression of *Bx-cyp29A3* increased with an increasing concentration after 24 h of L(−)-carvone exposure. The gene expression of *Bx-cyp29A3* reached its peak when the L(−)-carvone solution concentration was 0.8 mg/mL, which was 131.19 times higher than the control group. Interestingly, when the solution concentration reached 1.0 mg/mL, the gene expression of *Bx-cyp29A3* was similar to that at 0.4 mg/mL (Figure 5a). The gene expression of *Bx-cyp29A3* increased with an increasing concentration after 36 h of L(−)-carvone treatment. The gene expression of *Bx-cyp29A3* peaked when the L(−)-carvone solution concentration was 0.6 mg/mL, which was 27.37 times higher than that of the control group. Surprisingly, when the solution concentration reached 0.8 mg/mL, the gene expression of *Bx-cyp29A3* significantly decreased compared with the 24 h treatment group. However, the gene expression of *Bx-cyp29A3* remained similar to that of the 0.4 mg/mL treatment group when the solution concentration reached 1.0 mg/mL (Figure 5b).

**Figure 6:**
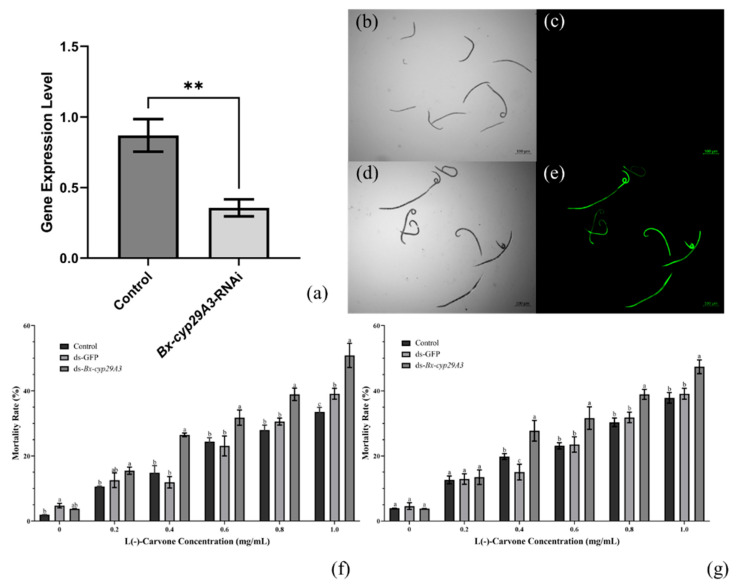
*Bx-cyp29A3* silencing response to L(−)-carvone stress. (**a**) Relative transcript level changes between dsRNA-treated and control group; (**b**–**e**) FITC-treated PWNs revealed green fluorescent signal under ultraviolet light; (**f**) the mortality rates of PWNs after treatment with L(−)-carvone and exposed for 24 h; (**g**) the mortality rates of PWNs after treatment with L(−)-carvone and exposed for 36 h. Notes: Scale bar = 100 μm. Data are mean values ± SD of different repetitions; *n* = 3. ** *p*-Value ≤ 0.01; different letters indicate statistical differences, with *p* ≤ 0.05 from Student’s *t*-test.

*Bx-cyp29A3* silencing was performed by soaking and the PWNs were detected by a microscopic examination without death during the soaking. qRT-PCR was then used to detect the efficiency of the *Bx-cyp29A3* silencing. The relative quantitative method calculation results, 2^−ΔΔCt^ = 0.3561 (*p* = 0.0024), indicated that *Bx-cyp29A3* was silenced and that the silencing efficiency was 64.39% (Figure 6a). FITC staining experiments demonstrated that exogenous substances could enter the nematode through the soaking method (Figure 6b–e).

L(−)-carvone has been found to regulate biological detoxification and immune responses via cytochrome P450 [63]. Thus, we investigated the resistance mechanism of *Bx-cyp29A3* by comparing the mortality of the PWNs treated with carvone and DEPC–H_2_O. The results of the tests showed that the mortality of the PWNs in the RNAi-treated group significantly increased at L(−)-carvone solution concentrations of 0.2, 0.4, 0.6, 0.8, and 1.0 mg/mL compared with that of the control group. After 24 h of treatment with 0.2, 0.4, 0.6, 0.8, and 1.0 mg/mL carvone solutions, the nematode mortality was elevated by 4.89%, 11.65%, 7.35%, 10.97%, and 17.27% in the RNAi-treated nematodes, respectively, compared with the control group (Figure 6f). After 36 h of treatment with 0.2, 0.4, 0.6, 0.8, and 1.0 mg/mL carvone solutions, the mortality rate in the RNAi-treated group increased by 0.81%, 7.91%, 8.51%, 8.56%, and 9.52%, respectively, compared with the control group (Figure 6g). The overall trend was that the *Bx-cyp29A3*-silenced group had a significantly higher mortality than the control group.

## 4. Discussion

PWNs are difficult to prevent and treat because they are widely distributed and rapidly spread. Since the discovery of PWD, many researchers have studied its pathogenesis and control methods, hoping to identify efficient and safe control measures. In this study, RNAi and bioinformatics analyses of *Bx-cyp29A3* were carried out at the molecular level. The present study was designed to determine the effect of *Bx-cyp29A3* silencing in response to exposure to potential nematocides. The sensitivity analysis of *Bx-cyp29A3* to different concentrations of L(−)-carvone provided an effective theoretical basis to reveal the metabolic mechanism and control measures for the detoxification of PWNs. Prior studies have proven that cytochrome P450 plays a crucial role in the low-temperature resistance, vitality, dispersal ability, reproduction, pathogenicity, and pesticide metabolism of PWNs [40,41,64,65]. However, to date, the mechanism of cytochrome P450 in the plant secondary metabolite resistance to PWNs remains unclear. In this study, the potential role of *Bx-cyp29A3* in the plant secondary metabolite resistance mechanism of PWNs was proven to be important for PWD. In biology, evolutionary trees are used to show the evolutionary relationships between species. The construction of evolutionary trees provides clues to predict the function of the target proteins. The evolutionary trees in this study indicated that *Bx-cyp29A3* was more similar to the *cyp* of plant-parasitic nematodes such as *A. avenae* and *D. destructor* than saprophytic nematodes and animal-parasitic nematodes. Protein structure and function analyses indicated that *Bx-cyp29A3* had a transmembrane structure and a molecular modification pocket that could modify chemicals and help to protect nematodes against an attack from hazardous materials.

The monoterpene carvone as a fragrance and flavor, growth inhibitor, antimicrobial agent, building block, and biochemical environmental indicator, along with its relevance in the medical field, attracts justifiable interest [66]. L(−)-carvone is a plant essential oil pesticide widely used against beetles such as *Reticulitermes dabieshanensis* [67], *Aromia bungii* [8], and *Callosobruchus chinensis* [68] that attack stored products (*Sitophilus oryzae*, *Rhyzopertha dominica*, and *Tribolium castaneum*) [69,70]. The results of this study found that the gene expression in response to 24 h L(−)-carvone solution exposure (0.2, 0.4, 0.6, 0.8, and 1.0 mg/mL) increased with the increasing concentration. When the solution concentration reached 0.8 mg/mL, the gene expression of *Bx-cyp29A3* peaked and was 131.19 times higher than that of the control group. The gene expression trend of the 36 h exposure to L(−)-carvone solutions (0.2, 0.4, 0.6, 0.8, and 1.0 mg/mL) was similar to that of the 24 h-treated groups. However, one interesting finding was that after 36 h of exposure, the expression peaked when the solution concentration reached 0.6 mg/mL and the gene expression of *Bx-cyp29A3* was 27.37 times higher than that of the control group. The results of this study indicated that *Bx-cyp29A3* could respond to exposure to multiple concentrations of a L(−)-carvone solution and the response at 24 h was superior to that at 36 h. The response increased with the increasing solution concentration and could appear in a shorter period. A possible explanation for this might be that cytochrome P450 is a phase I detoxifying enzyme in xenobiotic metabolism. The results of gene silencing revealed that *Bx-cyp29A3* silencing increased the sensitivity of PWNs to the L(−)-carvone solution. The results have a theoretical value for guiding the study of nematode xenobiotic metabolic mechanisms, colonization mechanisms, and molecular biological control. However, L(−)-carvone is a flavoring widely used to produce toothpaste [71] and chewing gum [72] or in biotransformation [73]. The production of carvone is extremely disruptive to the surrounding environment [74]. Therefore, the results of this study to evaluate the possible mechanisms of action and the safety of L(−)-carvone are essential.

As a plant essential oil, carvone positively affects PWNs and can be used as a potential nematocide. This is the first study of its impact on nematodes. The response increased with an increasing solution concentration and could appear in a shorter period. Importantly, we found that *Bx-cyp29A3* was a cytochrome P450 gene that responded to different concentrations of carvone. Gene silencing experiments clarified the function of *Bx*-*cyp29A3* in the immune defense of *B. xylophilus*. This is the basis for the action of cytochrome P450 in xenobiotic metabolism and provides a future target for the biological control of organisms. In the future, how to accurately control the concentration of L(−)-carvone to accurately control nematodes to avoid environmental pollution and nematocide waste must be studied.

## Figures and Tables

**Table 1:** Sequence homology comparison of *Bx-cyp29A3* with other species.

Scientific Name	Accession	Score	E-Value	Per. Ident
*D. destructor*	KAI1718381.1	379	3.00 × 10^−125^	39.69%
*A. avenae*	KAH7708527.1	373	4.00 × 10^−123^	43.78%
*Caenorhabditis brenneri*	EGT47445.1	348	1.00 × 10^−113^	38.45%
*C. remanei*	XP_003098490.1	345	2.00 × 10^−112^	39.48%
*C. briggsae*	XP_045095724.1	337	6.00 × 10^−109^	38.26%
*C. elegans*	NP_505490.2	333	1.00 × 10^−107^	38.81%
*Strongyloides ratti*	XP_024510577.1	321	7.00 × 10^−103^	37.30%
*Ancylostoma caninum*	RCN40602.1	308	6.00 × 10^−98^	37.23%
*Toxocara canis*	KHN86065.1	300	9.00 × 10^−95^	38.06%
*Pristionchus pacificus*	KAF8363934.1	293	6.00 × 10^−92^	34.84%

## Data Availability

Not applicable.

## Supplementary Materials

The following supporting information can be downloaded at: https://www.mdpi.com/article/10.3390/genes13111956/s1, Figure S1: Total RNA of PWNs and CDS region of *Bx-cyp29A3*. (a) Total RNA of PWN; (b) CDNA of PWN; (c) lane M DL2000 DNA marker; Lane 1: CDS of *Bx-cyp29A3*. Figure S2: The cDNA and deduced amino acid sequence of *Bx-cyp29A3*; Figure S3: Sequence alignment of cyp proteins in different species of nematode.
