# Supplementary material for: Functional Study on Cytochrome P450 in Response to L(−)-Carvone Stress in Bursaphelenchus xylophilus
Source: Genes (Basel). 2022 Oct 27;13(11):1956. doi: 10.3390/genes13111956 (PMC9689654; doi:10.3390/genes13111956)
Supplement: Supplementary file 1 [file genes-13-01956-s001.zip › genes-1981298-supplementary.pdf]

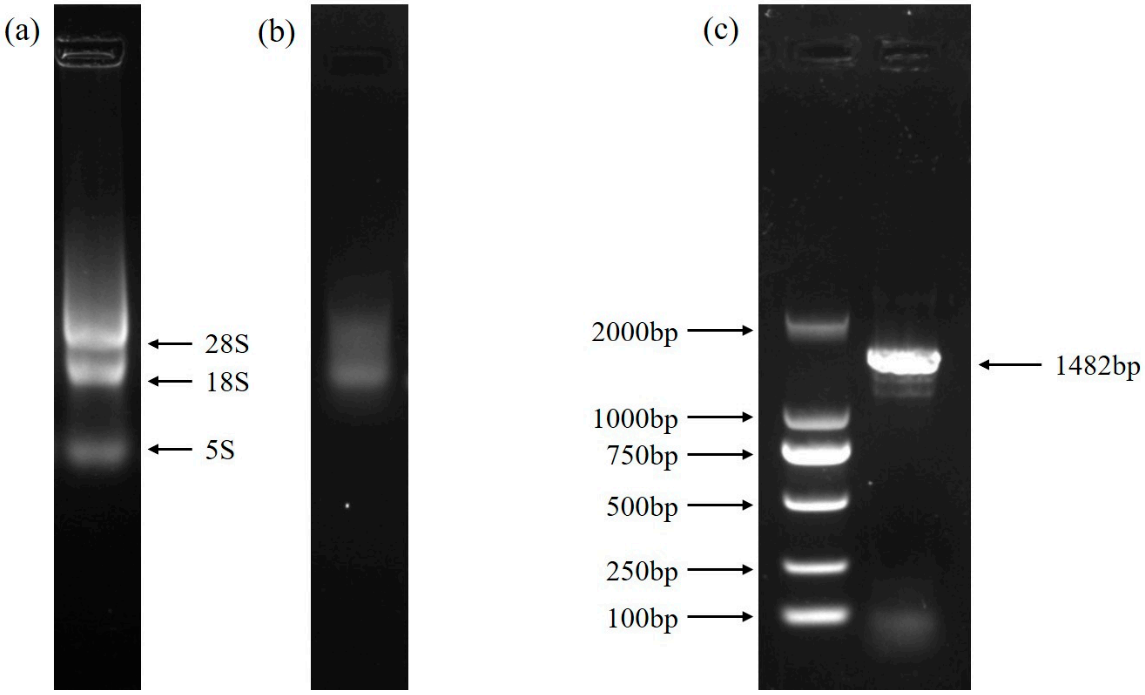

Figure S1. Total RNA of PWNs and CDS region of *Bx-cyp29A3*. (a) Total RNA of PWN; (b) CDNA of PWN; (c) lane M DL2000 DNA marker; Lane 1: CDS of *Bx-cyp29A3*.

>BXYJ5.010288100. cds: protein _ coding
ATGGCGTCGATTGTTGCGATTGCTTTGACTTTGATGTCTGCTTATATTGTGAATGCCATTCTGCCGGCGTTCTTGTTGTAT
ATTCGGCAGAAATGGATTGCGCGCAACTTGCCCGGCCCCAGAACAGACTTCTTCTTTGGCAACATTCTGCAATTCCCCA
AAGAATACAAAGATTATCGTGACTATTTCCTCGGCTTCGCCAACGAAGAGATTGCCAAAGGTCATAGTGTGGCGAGATT
ATGGGTTGCGAATCAGCTGTTTGTTTACCCGTTGAACAGCGACGCGACTCGCGCCATAACTTCCAGCACCGTCGAGCT
GAACAAGGGCGACGTCTACAGTTTTGTGGGACGTTGGCTGGGAAAGGGGCTCATCACGATCGGAAATGAGGAGCGAT
GGCATCGGAATCGCCGTCTTCTGACGCCGGCCTTTCATTTCGCAAAGCTGGACGAGTACATCCAGACTATTGATAGGCA
TGCAAGGCATTTGGTGCAAGTTATCCGCCAAAAATGCACCACTGGCCAAATTGACCTTTATCCGATAATCAAAATGTGT
GCGTTGGATGTGATTACCGACACCGCCATGGGCTGTCATATCAATGCGTTGGAGGACTCGGAACATCCGTACGTCAGAG
CCGTCCAGAAGTTCAACCATCTTATCTACATCAGAGCCATGAACCCGCTCTTATCCATATCGCTAGTTTGGACCTATTTGG
GGTATGAGAGGGAGAGCCAGAAGGCGTTGAAGGTGTTGAAGGACCAGACGTTTCAGGTGGTTACGGAGAGAATAAAA
CAACGCAAATTGGAGGACGCTAAGCCCCGGCCCGACTTCTTGGATCTGCTTTTGGATGAGTTTGACAAAGGAACGTTG
CCTTTTGAGGAAATCTGCGAAGAGGTGGACACATTCATGTTTGCCGGCCACGACACCACATCGCACGGTATCTCGTGG
ATCCTTTGGGCATTGGCTTGTCATCCCGAGGTTCAGGAGCAACTCTACGCCGAATTAATTGACAATTTCCCGAGCAAAG
AAGGTCTGTCGGCCATCAAGCAAAAGAATCTCAAATATTTGGATGCGGTTGTGAAAGAAGGACTTCGAATTTTCCCGG
CGGTTCCGTTTGTGCAAAGACGGTTGGTAAATGACTTGAGAATGGATGGGTACCTTATTCCGAGGGGCGCTCAAGTCA
CAATCGCACCATACTTTCTACATCACAATCCAAAGGTTTTTGCAAATCACATGGAGTTTGATCCGAACAACTTCTTGTCC
GGCAGGGACTACGAAAGCAATGCATATATTCCATTTAGCAGTGGGATGAGAAATTGTATTGGCCAACGATTTGCTCTGTA
CGAAATGAAAATCGTGGTTGCCTATTTGGTGTTGAACTTTACATTCTCGTCCGCCGGCCTCGGATTTCGCGAGAACTATC
CGACCTTTGAAGTCATCCTCCAGCCCAGTCATGGAGTCCCCGTCATTTTGGAAGAACGATGA

> BXYJ5.010288100 protein
MASIVAIALTLMSAYIVNAILPAFLLYIRQKWIARNLPGPRTDFFFGNILQFPKEYKDYRDYFLGFANEEIAKGHSVARLWVA
NQLFVYPLNSDATRAITSSTVELNKGDVYSFVGRWLGKGLITIGNEERWHRNRRLLTPAFHFAKLDEYIQTIDRHARHLVQV
IRQKCTTGQIDLYPIIKMCALDVITDTAMGCHINALEDSEHPYVRAVQKFNHLIYIRAMNPLLSISLVWTYLGYERESQKALK
VLKDQTFQVVTERIKQRKLEDAKPRPDFLDLLLDEFDKGTLPFEEICEEVDTFMFAGHDTTSHGISWILWALACHPEVQEQL
YAELIDNFPSKEGLSAIKQKNLKYLDAVVKEGLRIFPAVPFVQRRLVNDLRMDGYLIPRGAQVTIAPYFLHHNPKVFANHME
FDPNNFLSGRDYESNAYIPFSSGMRNCIGQRFALYEMKIVVAYLVLNFTFSSAGLGFRENYPTFEVILQPSHGVPVILEER

Figure S2. The cDNA and deduced amino acid sequence of *Bx-cyp29A3*

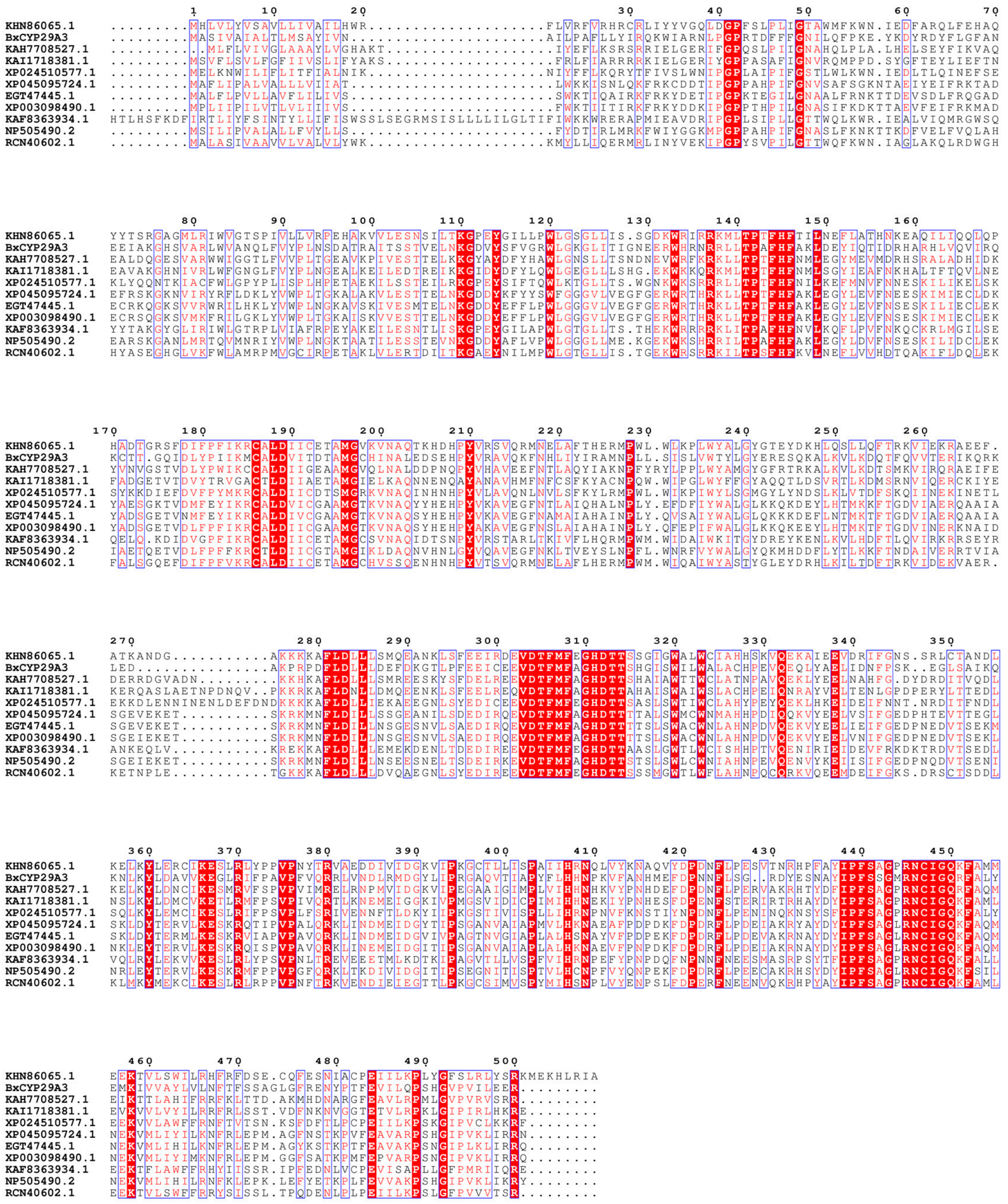

Figure S3. Sequence alignment of cyp proteins in different species of nematode
